# Effects of whole nutritional formula foods on nutritional improvement and intestinal flora in malnourished rats

**DOI:** 10.1002/fsn3.3865

**Published:** 2023-11-29

**Authors:** Di Qu, Pan‐Pan Bo, Zhi‐Man Li, Yin‐Shi Sun

**Affiliations:** ^1^ Institute of Special Animal and Plant Sciences Chinese Academy of Agricultural Sciences Changchun Jilin China; ^2^ Institute of Chinese Medicinal Materials Jilin Agricultural University Changchun Jilin China

**Keywords:** food for special medical purposes, ginseng water‐soluble dietary fiber, intestinal microecology, nutritional improvement, whole nutritional formula

## Abstract

Food for special medical purposes (FSMP) has received increasing attention as an enteral nutritional supplement. To investigate the effects of whole nutritional formula (WNF) containing dietary fiber and regular formula on nutritional supplementation and improvement of intestinal microecology, a rat malnutrition model was established with the formulations of WNF, FOS, and SDF (10, 20 g/kg bw) administered by gavage for 30 days. The results showed that the three formulations effectively improved the nutritional status of the malnourished rats, significantly increasing the level of IgG, increasing the abundance of *Bacteroidetes*, and affecting the content of propionic acid (PRO). The nutritional status of rats is closely related to growth performance, nutritional indexes, and immunoglobulin index, which cause changes in the composition of the intestinal flora. The above results showed that WNF positively affected the nutritional improvement, immune level, and intestinal health of rats. The comprehensive evaluation also suggested that the formulation containing ginseng water‐soluble dietary fiber (ginseng‐SDF) had the most significant effect.

## Graphical abstract

WNF foods improve the nutritional and immune factors level of malnourished rats. WNF foods has positive regulatory effect on SCFA and intestinal flora. WNF foods containing ginseng SDF have better improvement effect.

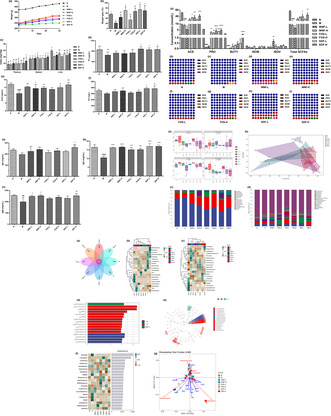

## 1 INTRODUCTION

Food for special medical purposes (FSMP) is designed to meet the special needs of patients with specific diseases for nutrients or diets. It can be divided into three categories: whole nutritional formula (WNF), specific whole nutritional formula (SWNF), and non‐whole nutritional formula (NWNF). With the aging of the population and the gradual increase of chronic diseases, the clinical application of FSMP has received more attention. Among these formulations, WNF can be used as a single source of nutrition to meet the nutritional needs of the target population.

Currently, most malnutrition occurs in patients after surgery or due to some diseases that lead to impaired absorption of gastrointestinal function. Treatment options include enteral and parenteral supplementation, but parenteral nutrition can only be used for a specific short period due to the single nutrient supplementation and the risk of causing decreased intestinal function and complications. Enteral nutrition supplementation has been a key research direction in recent years because of its high safety and efficacy. Malnutrition is closely related to dysbiosis of the intestinal flora. The intestinal microbiota composition is dynamic and influenced by the functional state of the dietary properties. Feeding intolerance (FI) is mainly caused by reduced postoperative gastrointestinal motility. Testing serum gastrointestinal hormone concentrations may be an important means of evaluating gastrointestinal function, but there is no universal method for effective postoperative monitoring (Melissas et al., 2013). Disease burden, surgical trauma, anesthesia, postoperative systemic inflammatory response, intestinal flora imbalance, and underlying disease may contribute to early FI. Early enteral nutrition (EN) can reduce the inflammatory response, nourish the intestinal mucosa, prevent bacterial translocation, and reduce postoperative infectious complications (He et al., 2022). Malnutrition from malignancy and therapeutic toxicity may reduce patient compliance with oncology treatment, thereby reducing treatment effectiveness. Furthermore, patients receiving nutritional support had a lower in‐hospital mortality rate than those not receiving nutritional support (Kaegi‐Braun et al., 2021).

Many studies have confirmed that dietary fiber has various physiological effects, such as hypolipidemic, hypoglycemic, antioxidant, and immune‐modulating effects (Deehan et al., 2022; Tawfick et al., 2021). Its regulatory effect on intestinal microecology has received much attention from scholars. Dietary fiber is divided into water‐soluble and water‐insoluble fibers, of which the water‐soluble dietary fiber can activate probiotics in the intestine and promote their growth and reproduction. A study targeting alcohol use disorder (AUD) showed that AUD patients treated with inulin had significantly improved social competence scores, increased serum brain‐derived neurotrophic factor levels, and modulated intestinal flora and social behavior (Amadieu et al., 2022). High dietary fiber intake significantly inhibited the inflammatory response in mice, altering the composition of intestinal flora in postnatal mice, reducing intestinal barrier damage, and facilitating the formation of microbial metabolite short‐chain fatty acids (SCFAs) (Liu et al., 2020). In addition, it is associated with a reduced risk of colorectal and breast cancers. The interaction between dietary nutrition and intestinal immunity is complex. In the process of reshaping the composition of the intestinal flora, dietary nutrients modulate the immunomodulatory function of immune cells in the intestine. In addition to promoting cecum health by modulating the antioxidant status of rats through intestinal probiotics, ginseng soluble dietary fiber (ginseng‐SDF) also increased the proportion of *Lactobacillus* and *Bifidobacterium* in the cecum flora (Hua et al., 2021).

This study investigated the nutritional improvement of conventional formula, fructo‐oligose formula (FOS), and ginseng‐SDF formula in malnourished rats. Their regulatory effect on the intestinal microecology of malnourished rats was also examined. This study can provide a basis for the practical application of FSMP in the future.

## 2 MATERIALS AND METHODS

### 2.1 Materials and formulations

The materials in this study are listed as follows: ginseng‐SDF, prepared according to the method of Hua et al. (2020); soybean isolate protein, maltodextrin (Wanbang Chemical Technology Co., Ltd); isolated whey protein (Mekaiwei Pharmaceutical Technology Co., Ltd); linseed oil powder (Hai Zhiyuan Life Science & Technology Co., Qingdao, China); medium chain triglyceride powder (MCT, Tianmei Biotechnology Co., Ltd); multivitamin, minerals (Shuowei Nutrition Technology Co., Guangzhou, China).

The three formulations are presented in Supplementary data S1.

### 2.2 Animals

The experimental animals were SPF male Wistar rats (8 weeks old, 180–200 g, qualification number of SCXK Liao 2020–0001), purchased from Changsheng Biotechnology Co., Ltd. Animal experiments were approved by the Laboratory Animal Management and Ethics Committee of the Institute of Special Animal and Plant Sciences, Chinese Academy of Agricultural Sciences (NO. ISAPSAEC‐2021–018).

### 2.3 Establishment of a *malnousished* rat model

A low‐nutrient rat model was established with reference to the method of Merino‐Sanjuan et al. A total of 48 rats were acclimatized and fed in an SPF‐grade environment at constant temperature and humidity for 7 days, and randomly divided into 8 groups (*n* = 6). All groups were fed with normal water (121°C, 15 min autoclave), and the normal group (N) was given 20 g/72.02 kcal/rat per day (72.7% carbohydrate, 12.5% protein, 4% fat), while the model group (M) was given 10 g/36.01 kcal/rat per day (58.2% carbohydrate, 10% protein, 3% fat). The rats were fed for 5 weeks and weighed daily. Body weight and serum albumin content were used as evaluation indicators, and the serum albumin content of the M group (17.53 ± 0.79 mg/mL) was significantly lower than that of the normal group (19.41 ± 0.82 mg/mL, *p* < .01). The body weight of rats in the M group (226.86 ± 23.81 g) was significantly lower than that of the N group (367 ± 9.92 g, *p* < .01), validating the effectiveness of the malnutrition rat model.

### 2.4 Feeding of WNF foods

The N and M groups continued to be fed according to the method in Section 2.3. The formula food group was given additional WNF food by gavage on top of the low‐nutrition feeding (10 g/36.01 kcal/animal). This group was further divided into 6 groups: WNF low‐dose group (WNF‐L), WNF high‐dose group (WNF‐H), FOS low‐dose group (FOS‐L), FOS high‐dose group (FOS‐H), ginseng‐SDF low‐dose group (SDF‐L), and ginseng‐SDF high‐dose group (SDF‐H). According to the dose conversion factor between rats and humans, the low‐dose group was set at 10 g/kg bw, and the high‐dose group was set at 20 g/kg bw. The feeding was administered by gavage once a day for 30 days.

### 2.5 Blood and tissue sample collection

After the final gavage, the rats were deprived of food and water for 24 h. Afterward, the rats were anesthetized with sodium pentobarbital (Cu Lai Bo Technology Co., Ltd). The hearts were bled and executed, the blood, liver, spleen, and thymus were removed, the organ weights were weighed, and the organ index was calculated [Organ index = organ weight (mg)/body weight (10 g)].

Anticoagulant was added to the blood in centrifuge tubes, mixed, and loaded into the machine. The test was conducted using a URIT‐2900 Vet Plus hematology analyzer (Unitech Electronics Group Co., Ltd). Blood was left at room temperature for 30 min, then centrifuged at 4°C (850 *g* for 30 min), and the serum was separated and stored at −80°C for biochemical analysis.

### 2.6 Determination of biochemical indexes

The determination of biochemical indexes in serum was performed according to the instructions provided in the kit. The indexes include serum albumin (ALB), serum prealbumin (PA), total cholesterol (TC), Immunoglobulin A (IgA), immunoglobulin G (IgG), immunoglobulin M (IgM) (Enzyme‐linked Biotechnology Co., Ltd), and total protein (Rui Da Heng Hui Technology Development Co., Ltd).

### 2.7 Fecal collection

All rats were deprived of water for 12 h after the last dose, and two copies of rat feces (one for the determination of short‐chain fatty acids and one for 16S rRNA sequencing) were collected aseptically and stored at −80°C.

### 2.8 Determination of SCFAs

Short‐chain fatty acids (SCFAs) were determined with reference to Inoue et al. (2019) with slight modifications. Gradient concentration standard solutions of acetic acid (ACE), propionic acid (PRO), butyric acid (BUTY), isobutyric acid (ISOB), valeric acid (VAL), and isovaleric acid (ISOV) were prepared to make the standard curve. A certain mass of rat feces was added to deionized water in the ratio of 1:3 (W/V), vortexed and mixed, and extracted at 4°C for 12 h, followed by centrifugation at 8,000 *g* for 10 min (4°C). The supernatant was aspirated to 200 μL, and 40 μL of a solution containing 2 g/L internal standard 2‐ethylbutyric acids (TE007025ML, Sinopharm Chemical Reagent Co., Ltd., dissolved in 25% metaphosphoric acid) was added. After vortexing and mixing, the mixture was placed in an ice bath for 35 min, followed by centrifugation at 10,000 *g* for 10 min (4°C). DB‐FFAP column (30 m × 0.32 mm × 0.32 μm) with a nitrogen carrier gas at a flow rate of 2.2 mL/min and an FID detector was used. The injection temperature was 250°C, and the initial temperature was 60°C, which ramped up to 170°C at 10°C/min and then to 212°C at 8°C/min.

### 2.9 Determination of intestinal microorganisms

Approximately 0.25 g of feces was preserved in dry ice and sent to Shanghai Private Biotechnology Co., Ltd. for gut microbial 16S rRNA detection. Microbial genomic DNA was extracted from the samples using the QIAamp DNA Fecal Mini Kit (No. 51504, QIAGEN China (Shanghai) Co., Ltd) according to the manufacturer's instructions, and the quality inspection of DNA samples was performed by agarose gel electrophoresis. Polymerase chain reaction amplification primers (forward primer: 5′‐ACTCCTACGGGAGGCAGCA‐3′, reverse primer: 5′‐CGGACTACHVGGGTWTCTAAT‐3′) were designed by selecting the 16S rRNA V3‐V4 region. Paired‐end sequencing was performed using the Illumina NovaSeq platform, and the obtained sequences were denoised, merged, and non‐chimerized using the DADA2 method of QIIME 2 software. The sequencing data were classified into different OTUs according to a 97% similarity threshold. The Genescloud platform (https://www.genescloud.cn) was used for correlation analysis. For taxonomic composition analysis, which was performed on a feature table after removing singletons, the composition distribution of each sample was visualized at the six classification levels. R language and the Venn diagram package were used to draw Venn diagrams of common/unique OTUs between different groups (https://en.wikipedia.org/wiki/Venn_diagram). R language and the PheatMap package were used to calculate the clustering results of each sample and each taxon, and the results of the species composition heat map were presented in the form of an interactive graph. Correlations between the microbial community and physiological indicators were analyzed by redundancy analysis (RDA), which was performed by Genescloud tools, a free online platform for data analysis (https://www.genescloud.cn) from Shanghai Personal Biotechnology Co., Ltd.

### 2.10 Data processing

Data were presented as mean ± SD (*n* = 6), and the ANOVA test was performed using SPSS 24.0. The results were plotted using GraphPad Prism 8.0.

## 3 RESULTS

### 3.1 Evaluation of nutritional improvement in rats fed with WNF foods

**FIGURE 1:**
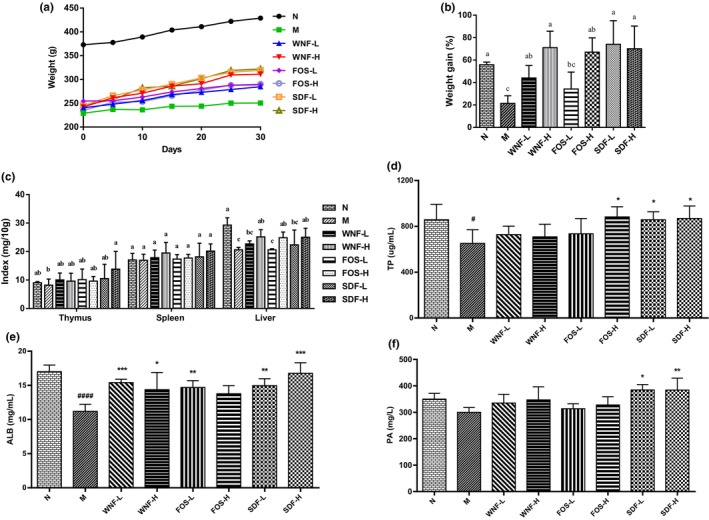
Effect of WNF on growth performance and nutritional indexes of rats. (a) the body weight of rats after 30 days of gavage; (b) body weight growth rate of rats after 30 days of gavage; (c) thymus, spleen, and liver indexes; (d) TP content; (e) ALB content; (f) PA content. Results are significant when #*p* < .05, ####*p* < .0001 (compared between the N and M groups), *p < .05, **p < .01, ***p < .001 (compared with the M group).

The rats in all groups were in good physical condition during the 30‐day feeding and showed a gradual increase in body weight (Figure 1a). With the end of feeding, the growth rate of body mass of rats in group M was significantly lower than group N (*p* < .05), there was no significant change in WNF‐l and FOS‐L groups compared with group M, and the growth rate of body mass of rats in WNF‐H, FOS‐H, SDF‐L, and SDF‐H groups was significantly higher than that in group M (*p* < .05; Figure 1b). The results showed that, whatever the formula, the high dose group can significantly increase the growth rate of body mass in rats, and the SDF‐L group can also significantly increase the growth rate of body mass, indicating that SDF has a good nutritional supplement effect. The results of the visceral index showed that (Figure 1c) the thymus index in group M was significantly lower than group N, and only SDF‐H was significantly higher than group M. There was no significant change in the spleen index. The liver index in group M was significantly lower than that in group N, and the WNF‐H, FOS‐H, and SDF‐H groups could significantly improve the liver index.

Total serum protein is one of the important indicators for evaluating malnutrition. Figure 1d shows that the TP content of rats in the M group was significantly lower than that of the N group (*p* < .05), and the rats in the FOS‐H, SDF‐L, and SDF‐H groups had significantly increased TP content after gavage, indicating that FOS and ginseng‐SDF assisted protein absorption. ALB is the most abundant protein in the blood, providing nutritional support to tissues and enabling antioxidants, transport, and detoxification. Its level decreases in states of inflammation and malnutrition. As shown in Figure 1e, the ALB level in group M was significantly lower than that in group N (*p* < .0001), and ALB level in all experimental groups except group FOS‐H was increased to varying degrees. ALB in the WNF‐H, FOS‐L, and SDF‐L groups was significantly increased (*p* < .05, *p* < .01), and it was extremely significantly increased in the WNF‐L and SDF‐H groups (*p* < .001). The PA detection results showed that the PA level in the SDF‐L and SDF‐H groups was significantly higher than that in the M group (*p* < .05, *p* < .01; Figure 1f). It can be concluded that the whole nutrition formula food containing ginseng‐SDF can significantly improve the growth performance and nutritional status of malnourished rats.

### 3.2 Effect of WNF foods on blood indexes and immune factors in rats

As shown in Table 1, the research group tested RBC, Hb, LYM, WBC, and NEU indexes in the blood of rats, which could be used as preliminary indexes for evaluating immune function. The indexes of group M were lower than those of group N, but the difference was not significant (*p* > .05). The RBC content in FOS‐L and SDF‐L groups was significantly increased (*p* < .05), the Hb content in FOS‐L, FOS‐H, and SDF‐H groups was significantly increased (*p* < .05), and the LYM content in the FOS‐H group was significantly increased (*p* < .05). The contents of WBC and NEU in the SDF‐H group were significantly increased (*p* < .05).

**TABLE 1:** Effects of three kinds of WNF foods on the blood indexes of malnourished rats.

Groups	RBC (10^12/L)	Hb (g/dL)	LYM (10^9/L)	WBC (10^9/L)	NEU (10^9/L)
N	5.99 ± 1.53^abc^	106 ± 24.04^abc^	1.44 ± 0.77^ab^	3 ± 1.07^b^	0.42 ± 0.27^b^
M	4.83 ± 1.01^c^	84.25 ± 20.43^c^	1.24 ± 0.96^ab^	2.33 ± 0.91^b^	0.27 ± 0.09^b^
WNF‐L	4.95 ± 1.66^bc^	91 ± 33.94^bc^	0.46 ± 0.21^b^	2.7 ± 0.22^b^	0.3 ± 0.17^b^
WNF‐H	6.02 ± 0.82^abc^	110.8 ± 16.97^abc^	1.38 ± 0.61^ab^	4.1 ± 1.65^ab^	1.36 ± 1.19^ab^
FOS‐L	7.05 ± 0.40^a^	118 ± 6.04^ab^	1.3 ± 0.62^ab^	2.65 ± 0.64^b^	0.48 ± 0.16^b^
FOS‐H	6.51 ± 0.67^abc^	130.67 ± 8.09^a^	1.67 ± 1.09^a^	3.68 ± 1.01^ab^	1.42 ± 1.044^ab^
SDF‐L	6.72 ± 0.38^ab^	112.5 ± 6.80^abc^	1.05 ± 0.54^ab^	2.55 ± 0.63^b^	0.45 ± 0.13^b^
SDF‐H	6.01 ± 1.09^abc^	126 ± 10.16^a^	1.5 ± 1.32^ab^	5.4 ± 1.77^a^	2.06 ± 1.27^a^

*Note*: The data are means±SD (*n* = 6). Different letters in the same row indicate a significant difference (*p* < .05).

**FIGURE 2:**
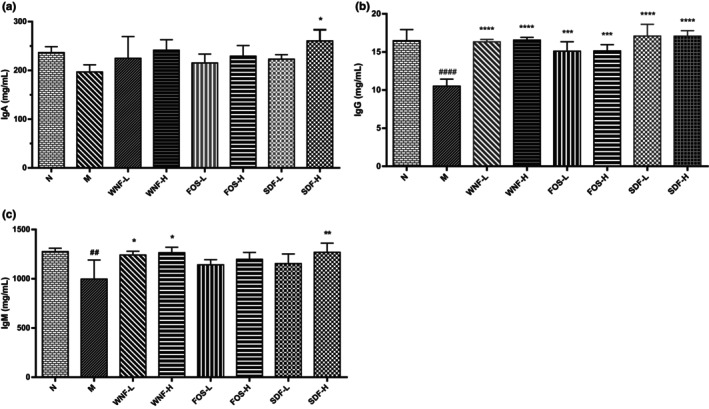
Effect of WNF foods on immunoglobulins in rats. (a) IgA; (b) IgG; (c) IgM. Results are significant when ##*p* < .01, ####*p* < .0001 (compared only between the N and M groups), *p < .05, **p < .01, ****p < .0001 (compared with the M group).

Immunoglobulins are closely related to the innate immunity, proliferation, and migration of tumor cells. IgA, IgG, and IgM are important immune factors for early defense. Malnourished patients often exhibit compromised immune function, which can lead to a variety of diseases. As shown in Figure 2, the IgA content in the SDF‐H group was significantly higher than that in the M group (*p* < .05), and there was no significant change in the other groups. The IgG content of group M was significantly lower than that of the N group (*p* < .0001), and the IgG content of group M was significantly increased by all formula groups (*p* < .0001). The IgM content in group M was significantly lower than that in group N (*p* < .01), and that in the WNF‐L, WNF‐H, and SDF‐H groups was significantly higher than that in group M (*p* < .05, *p* < .01).

### 3.3 Effect of WNF foods on the intestinal health of rats

#### 3.3.1 Effect of WNF foods on SCFAs in the intestines of rats

**FIGURE 3:**
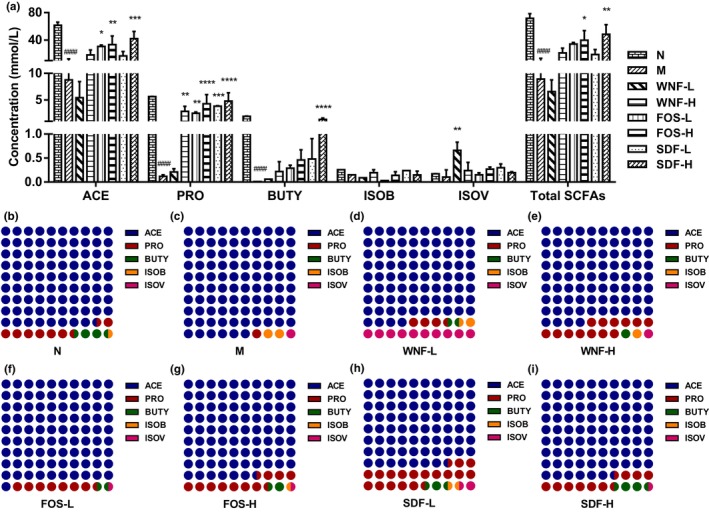
Effect of WNF foods on content (a) and ratio (b‐i) of fecal SCFAs in rats.

SCFAs are metabolic by‐products of the microbial fermentation of complex polysaccharides. They are undigested or partially digested in the human small intestine and play a critical role in maintaining intestinal health and the morphology and function of the colon epithelium. Previous studies have shown that SCFAs can affect tissues and organs beyond the gut through the bloodstream, with a strong link to liver health, diabetes, and metabolic diseases associated with obesity (van der Hee & Wells, 2021). In this study, as shown in Figure 3a, total SCFAs in group M were significantly lower than those in group N (*p* < .001), and total SCFAs in groups FOS‐H and SDF‐H were significantly increased after gavage (*p* < .05). ACE, PRO, and BUTY were the major SCFAs, and the M group was significantly lower than the N group (*p* < .0001), indicating that malnutrition had a significant effect on intestinal health. ACE was significantly higher in the FOS‐L, FOS‐H, and SDF‐H groups compared to the M group. PRO was significantly higher in all groups except the WNF‐L group, with highly significant increases in the FOS‐H and SDF‐H groups (*p* < .0001). Only the SDF‐H group was extremely significantly higher than the M group in BUTY (*p* < .0001); there was no significant difference among other groups (*p* > .05). The composition of SCFAs differed greatly between groups. Compared with group N, the ACE of group M increased from 88.49% to 95.8%, the PRO decreased from 8.09% to 1.31%, and the BUTY decreased from 2.8% to 0.1%. After gavage, the content of PRO and BUTY increased to different degrees compared to the M group, with the most significant increase in BUTY in the SDF‐L and SDF‐H groups. Previous studies have shown that ginseng‐SDF can promote the production of BUTY in cecum contents and have prebiotic properties (Hua et al., 2021).

#### 3.3.2 Analysis of the structure and composition of rat fecal flora

**FIGURE 4:**
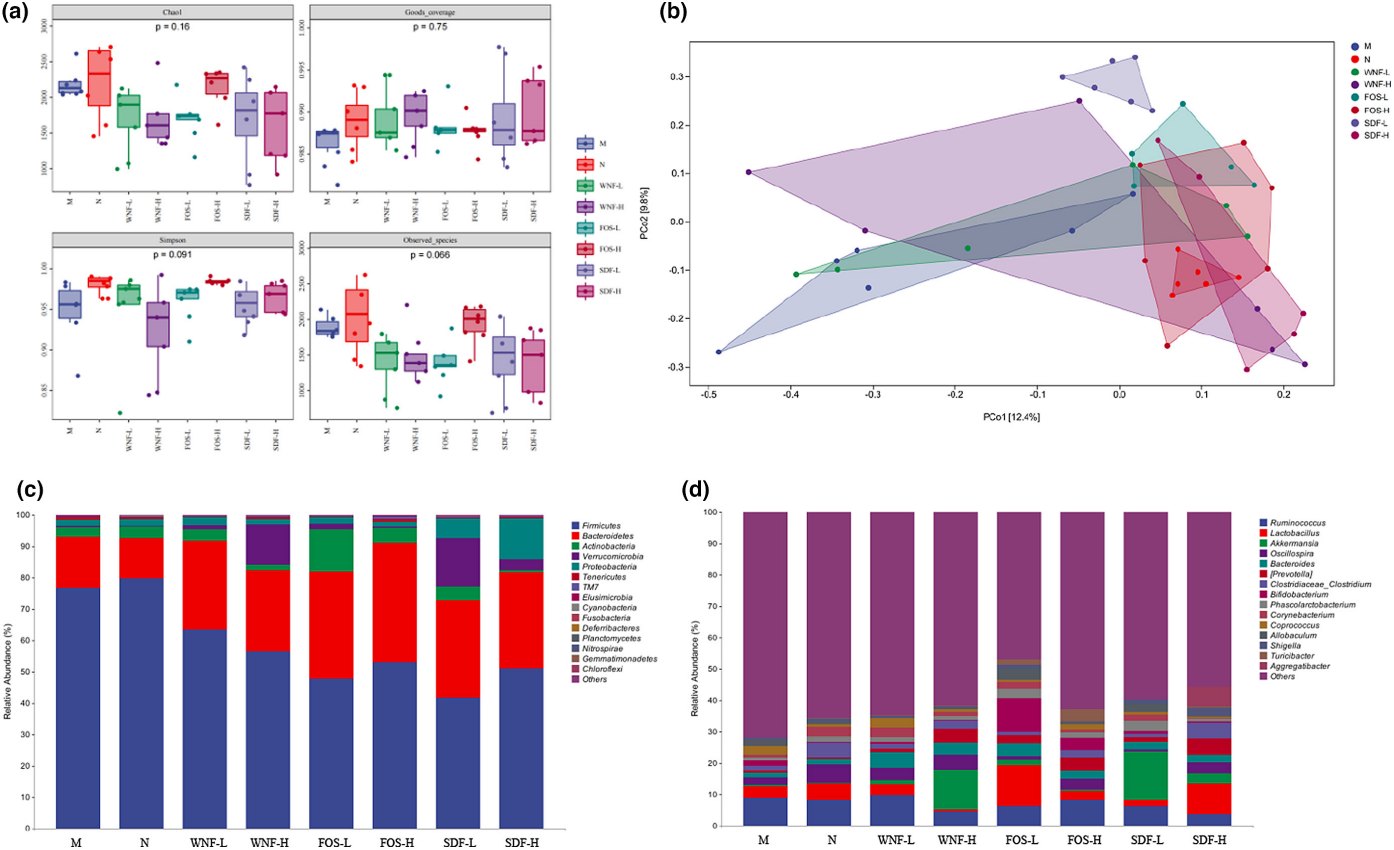
Effect of WNF foods on intestinal microbial diversity and flora composition in rats. (a) rat intestinal microbial diversity analysis; (b) PCoA analysis; (c) taxonomic composition at the phylum level; (d) taxonomic composition at the genus level.

The α‐diversity analysis is shown in Figure 4a. Compared with group N, the richness indices Chao1 and observed species were significantly lower in group M, but the difference was insignificant; the FOS‐H group was significantly higher and close to the normal group. The Simpson diversity index was particularly sensitive to the dominant OTU in the community. Compared to the N group, the Simpson index was significantly lower in the M group, but the difference was insignificant. After gavage, the FOS‐H group was significantly higher compared to the M group (*p* < .05), and there was no significant difference in the coverage index Good (*p* > .05). The PCoA analysis is shown in Figure 4b. The sample points of the N and WNF‐H, FOS‐H, and SDF‐H groups overlapped more with the M, WNF‐L, FOS‐L, and SDF‐L groups, while these sample points had no overlap with the M, WNF‐L, FOS‐L, and SDF‐L groups.

At the phylum level (Figure 4c), *Firmicutes*, *Bacteroidetes*, *Actinobacteria*, *Verrucomicrobia*, and *Proteobacteria* are the main components of the rat intestinal flora. *Firmicutes* have the highest relative abundance, followed by *Bacteroidetes*, making them dominant colonies at the phylum level. As the metabolite of *Firmicutes*, BUTY has anti‐inflammatory and insulin resistance effects, making it effective in preventing obesity and type II diabetes (Everard et al., 2014). It also ferments various carbohydrates, participates in the metabolism of bile acids, and maintains intestinal health (Shang et al., 2017). Compared to the M group, the *Firmicutes* of FOS‐L, FOS‐H, and SDF‐L were significantly lower (*p* < .05), and the relative abundance of *Bacteroidetes* increased in all groups.

At the genus level (Figure 4d), the M group had the lowest abundance levels, with *Ruminococcus*, *Lactobacillus*, *Akkermansia*, *Oscillospira*, and *Bacteroides* dominating the intestinal flora of the rats. Among them, *Lactobacillus* is a recognized beneficial bacterium with the ability to regulate intestinal immunity, maintain intestinal health, and regulate intestinal disorders (Wang et al., 2017). Compared with the K group, the M group reduced the relative abundance of *Oscillospira*, *Lactobacillus*, *Clostridiaceae_Clostridium*, and *Phascolarctobacterium*. Compared with the M group, the relative abundance of *Lactobacillus* was up‐regulated in the FOS‐L and SDF‐H groups. In the WNF‐H and SDF‐L groups, the relative abundance of *Akkermansia* increased, and the relative abundance of *Ruminococcus* and *Lactobacillus* decreased. In addition, *Ruminococcus* and *Lactobacillus* belong to the same group of *Firmicutes*, which hydrolyze amylase and sugars to produce butyrate and SCFAs (Louis & Flint, 2009).

#### 3.3.3 Marker species analysis of the intestinal flora

**FIGURE 5:**
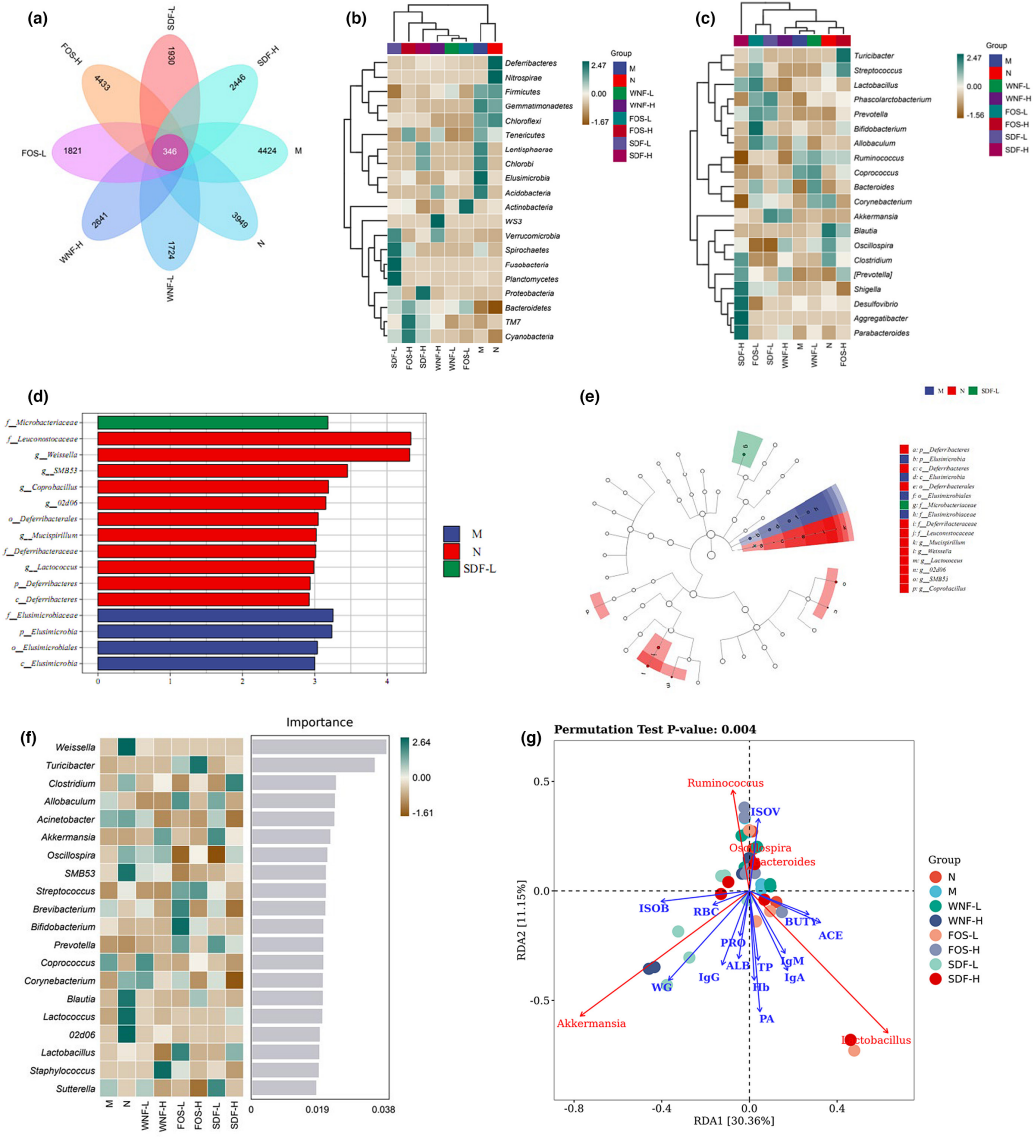
Effect of WNF foods on the characteristics of rat intestinal flora. (a) Venn diagram of OTUs in each group; (b) heat map at the phylum level; (c) heat map at the genus level; (d and e) LEfse analysis between groups; (f) random forest heat map at the genus level; (g) RDA analysis of major genera with growth performance, immune factors, and SCFAs; blue arrows represent indicators or factors; red arrows represent flora; and different dots represent different samples.

A Venn diagram was created based on the number of common and unique OTUs for each sample (Figure 5a). The figure shows that the number of OTUs in each group was N (3949), M (4424), WNF‐L (1724), WNF‐H (2641), FOS‐L (1821), FOS‐H (4433), SDF‐L (1930), and SDF‐H (2446), with a total of 346 OTUs in each of the eight groups and a large variation in flora between groups.

The species composition heat map shows that at the phylum level (Figure 5b), *Deferribacteres*, *Nitrospirae*, *Firmicutes*, *Gemmatimonadetes*, and *Chloroflexi* have higher response values in the N and M groups. The values of *Deferribacteres* and *Nitrospirae* decreased, and *Tenericutes*, *Lentisphaerae*, *Chlorobi*, *Elusimicrobia*, and *Acidobacteria* increased. Compared to the M group, the SDF‐L group had significantly higher values of *Verrucomicrobia*, *Spirochaetes*, *Fusobacteria*, and *Planctomycetes*. The FOS‐H and SDF‐H groups increased the response values of *Bacteroidetes*, *TM7*, and *Cyanobacteria*. At the genus level (Figure 5c), the response values were higher for *Ruminococcus*, *Corynebacterium*, *Blautia*, *Oscillospira*, and *Clostridium* in the N group. *Allobaculum*, *Ruminococcus*, and *Coprococcus* had higher response values in the M group. All other genera were at lower levels compared to the M group. FOS‐L and SDF‐H groups had a greater effect on the genera. *Shigella*, *Desulfovibrio*, *Aggregatibacter*, *Parabacteroides*, *Lactobacillus*, and *Bifidobacterium* were all elevated to varying degrees.

Further LEfse analysis (Figure 5d) filtered out biomarkers with statistically significant differences, with 11 markers (such as *Leuconostocaceae* and *Weissella et al.*) in group N, four iconic species (such as *Elusimicrobiaceae et al.*) in group M, there is only one species of *Microbacteriaceae* in group SDF‐L. And no significant differences were found in the other groups.

A random forest model was used to further analyze the main flora responsible for the differences in rat fecal flora between the WNF, N, and M groups. The heat map is shown in Figure 5e. *Weissella* and *Turicibacter* were the two most iconic species at the genus level. *Weissella*, *SMB53*, *Blautia*, *Lactococcus*, and *02d06* were the most abundant genera in the N group. There was a significant decrease in several important strains and a significant increase in the abundance of *Coprococcus* in the M group, which is considered a dysbiosis of gut flora in malnourished children (Smith et al., 2013). Compared to the M group, the FOS‐L group had the greatest effect on the flora, increasing the abundance of *Lactobacillus* and *Bifidobacterium*. Except for the WNF‐L group, the abundance of *Coprococcus* decreased in the WNF groups. The changes in the abundance of these genera were the main reasons for the differences in the structure of the rat fecal flora between the M and WNF groups.

RDA analysis was performed on physiological indicators and the genus‐level flora of rats (Figure 5f). PA and WG had the greatest effect on the intestinal flora; *Ruminococcus* was positively correlated with ISOV; *Akkermansia* and *Lactobacillus* were positively correlated with IgM, IgA, TP, Hb, PA, IgG, ALB, PRO, and WG factors. These results indicate that WNF foods may regulate physiological indicators in rats by modulating the intestinal flora.

## 4 DISCUSSION

### 4.1 Nutritional improvement in rats with WNF foods

This study showed that all three WNF foods improved the nutritional status of malnourished rats, with the WNF foods containing ginseng‐SDF having the most significant nutritional improvement in rats. Previous studies have shown that intestinal nutrition supplementation can satisfy the caloric and protein intakes of rats (Campos‐Martinez et al., 2022). The WNF foods in this study had a good amino acid distribution with high arginine and lysine content, making them a good source of protein (de Aguilar‐Nascimento et al., 2011). In addition, whey protein has good palatability and digestibility, providing a large amount of branched‐chain amino acids, especially leucine (García‐Talavera Espín et al., 2010). Ginseng‐SDF can significantly improve growth performance and energy absorption in rats (Hua et al., 2021), which is the main reason for increased body weight and serum nutritional indexes after gavage. As soluble dietary fiber can be fermented by colonic bacteria to produce SCFAs (especially BUTY), adding dietary fiber to enteral nutrition formulations has become essential. Compared with the rats in the M group, those fed with ginseng‐SDF foods had higher ACE, PRO, and BUTY in the feces. The BUTY was significantly higher in those rats. SCFAs are a key nutrient for the intestinal epithelium, and formulations containing SDF are beneficial (Pekmez et al., 2019). Gastrointestinal peptides involved in appetite and metabolic regulation are also influenced by the intestinal flora. By influencing SCFAs, the intestinal flora is involved in the metabolic regulation of the organism and may also influence the host appetite and metabolism through its relationship with other appetite‐regulating hormones (e.g., leptin) and pro‐appetite hormones (e.g., ghrelin) (Queipo‐Ortuño et al., 2013). Metabolites of probiotics, such as butyrate, can reduce the atrophy of the intestinal wall caused by malnutrition and restore nutritional status (Martínez‐Cerón & Álvarez‐Sala, 2012). Probiotics can also help maintain a healthy physiological environment, correct ecological disorders, and improve nutrient malabsorption.

In this study, the liver index was significantly lower (*p* < .05) in malnourished rats compared to the N group. The high‐dose group of each formula significantly increased the liver index after feeding with the WNF foods (*p* < .05). The liver is an important organ for the metabolism of protein, fat, and carbohydrates. Because liver health is closely related to malnutrition (Li et al., 2021), healthy internal organs are the basis for maintaining the good growth of the organism. Adejuwon has reported that rats fed with good fermented and unfermented sorghum, soybeans, and orange‐fleshed sweet potatoes can provide a high‐quality source of protein, carbohydrate, and oil, and improve liver index (Adejuwon et al., 2013).

### 4.2 Immunomodulatory effects of WNF foods in rats

Several studies have shown that giving patients reasonable and effective nutritional support can promote the balance of energy required by the body, ameliorate malnutrition, maintain the daily supply of nutrients required by the body tissues, and further improve immune function (Bhatt et al., 2017). IgM is the first immunoglobulin produced in the initial immune response, accounting for 5%–10% of the total immunoglobulins in the serum. As the main component of serum, IgG accounts for about 75% of the total immunoglobulin content. It has viral neutralizing, antibacterial, and immunomodulatory functions, and its expression level is directly proportional to the body's resistance. In this study, the levels of IgM and IgG in *malnourished* rats were significantly lower than those in normal rats (*p* < .01, *p* < .001), indicating that nutritional deficiency can significantly reduce the immune function of the body. The IgG content significantly increased (*p* < .001) after supplementation with WNF foods. The results of this study showed that IgG was significantly positively correlated with Akkermansia, Lactobacillus, and PRO. SCFAs are an important source of energy for the epithelial cells of the colonic mucosa, which can enhance the integrity of the mechanical barrier of the intestinal mucosa and strengthen autoimmunity. In addition, the thymus is an important lymphoid organ closely related to immunity. This study confirmed that malnutrition could lead to a significant decrease in the thymus index in rats (*p* < .05), and SDF‐H could significantly improve the thymus index (*p* < .05). Hua et al. (2021) showed that ginseng‐SDF has a good immunomodulatory function. It affects the directional relationship between the intestine and immunity by regulating the intestinal flora and influencing the bidirectional communication between the intestine and immunity.

Nutritional supplementation is key to enhancing immunity, and supplementation with appropriate amounts of micro‐ and macronutrients can significantly improve immune‐related indicators. Nutritional therapy for patients with severe COVID‐19 improves immunity and prevents virus spread (Calcuttawala, 2022). The importance of FSMP for enteral nutrition has been widely recognized by the industry for a long time (Schütz et al., 2006). Administration of FSMP can facilitate recovery by preventing disease progression, reducing side effects from medications, decreasing pain and complications, and shortening the length of hospital stay (Wang et al., 2022). de Oliveira et al. (2014) investigated the mechanisms by which signaling pathways regulate the immune system under malnutrition conditions. The results showed that the expression of phosphorylated NF‐ĸB (important for normal macrophage function) was lower in malnourished mice than in normally fed mice. In ATP, protein kinase acts as a phosphate donor, binding a hydroxyl group to a serine, threonine, or tyrosine residue of the target protein for post‐translational modification of phosphorylation (Yang & Terman, 2017). Similarly, all energy required for cellular processes is obtained from ATP (Ledderose et al., 2014). The results obtained by de Oliveira et al. (2014) could be due to inadequate protein intake in the experimental group, as protein intake is important for delivering ATP to body organs and providing adequate energy. Therefore, supplementation with WNF foods can be effective in improving the immunity and resistance of the body.

### 4.3 Regulation of intestinal flora in rats by WNF foods

Gut microbial cells play an important role in maintaining local and systemic homeostasis by interacting with the immune, endocrine, and nervous systems of the body (Lynch & Pedersen, 2016). Healthy intestinal microbes have high diversity, structural complexity, and stability. They can resist external environmental stresses and maintain a stable flora structure under external disturbances. In contrast, non‐healthy humans exhibit reduced flora diversity, loss of resistance to external environmental stresses, and adverse conditions such as intestinal microecological disorders (Dong et al., 2022). In this study, the nutritional improvement significantly enhanced the intestinal flora of rats, with increased levels of ACE, PRO, and BUTY in rat feces. The effects of probiotics can be mediated through their metabolites with anti‐inflammatory effects, such as SCFAs (particularly PRO, ACE, and BUTY). These postbiotics function by binding to specific receptors on intestinal epithelial cells. SCFAs provide approximately 10% of the total energy supply in a human diet.

The intestinal flora is a microbial ecosystem that plays an important role in human health. Several characteristics are now available to determine the health of the gut flora, including diversity of the flora, genetic richness, number of butyrate‐producing species, and resilience. In addition to activating the growth and development of the immune response, gut flora also plays a crucial role in the maturation of immune cells. The diversity and abundance of gut microbes are considered powerful determinants of host health, and changes in diversity have been associated with several human diseases. However, several studies have shown that the gut flora is directly involved in the pathophysiological processes of specific diseases through complex interactions between the gut flora, host metabolism, and the immune system (Yang et al., 2005). Gut flora dysbiosis may be a cause or a consequence of mucosal inflammation. The present study confirmed that intestinal flora diversity and richness were significantly increased in rats fed the FOS‐H group. *Firmicutes* and *Bacteroidetes* are the two dominant flora in the rat intestine, with *Firmicutes* being a source of several SCFAs (Murugesan et al., 2018). It has also been reported that the ratio of *Firmicutes* to *Bacteroidetes* (F/B) is considered an indicator of gut microbiota and is strongly associated with body weight, body fat, and obesity (Magne et al., 2020). Both are among the most abundant taxa of the intestinal microbiota, and their dynamics are highly significant despite significant inter‐individual differences (Mbakwa et al., 2018). The results of this study suggest that WNF foods can effectively increase the production of beneficial bacteria in the intestinal flora of rats and improve intestinal health.

In this experiment, the N, M, and SDF‐L groups showed their signature species. *Leuconostocaceae* and *Weissella* are the main markers of the N group and important genera in the intestinal flora, with antipathogenic and significant prebiotic properties. *Leuconostocaceae* have high gastrointestinal tolerance and antagonistic activity against foodborne pathogenic bacteria. It can also enhance SCFA production by adhering to HT‐29 cells (Matsuzaki et al., 2018). *Weissella* has been demonstrated to have various physiological functions, including intestinal homeostasis control, immunomodulation, pathogenic bacterial inhibition, anti‐allergy, anti‐tumor, and aging delay (Leroy & De Vuyst, 2004). These signature species are key factors in maintaining the health and stability of normal rat intestinal microecology.

## 5 CONCLUSION

This study investigated the effects of WNF foods on the improvement of malnutrition and the intestinal flora of rats in terms of growth performance, immune factor levels, and intestinal microbial composition. The results showed that the WNF foods increased the body weight, Hb, and RBC content of rats, and the WNF foods containing ginseng‐SDF increased the serum TP, ALB, and PA levels. Compared to the M group, the SDF‐H group had higher thymic and liver indexes, and WNF significantly increased immunoglobulins, especially IgG. The intestinal flora structure of rats fed WNF foods was significantly altered. The abundance of *Bacteroidetes* was increased, the abundance of *Firmicutes* was decreased, and the concentration of SCFAs in the feces was increased (especially PRO). The most significant changes in SCFAs were observed in the SDF‐H group, especially for BUTY. *Ruminococcus* was positively correlated with ISOV, and *Akkermansia* and *Lactobacillus* were positively correlated with IgM, IgA, TP, Hb, PA, IgG, ALB, PRO, and WG factors. The above results indicated that WNF foods could effectively improve the nutritional status of malnourished rats, enhance immunity, and improve intestinal microecology. The comprehensive evaluation also suggested that the formula containing ginseng‐SDF had the most significant effect.

## AUTHOR CONTRIBUTIONS

**Di Qu:** Funding acquisition (equal); resources (equal); writing – original draft (equal); writing – review and editing (equal). **Pan‐Pan Bo:** Formal analysis (equal); methodology (equal); project administration (equal). **Zhi‐Man Li:** Validation (equal); visualization (equal). **Yin‐Shi Sun:** Funding acquisition (equal); investigation (equal); supervision (equal).

## FUNDING INFORMATION

This work was supported by the Scientific and Technology Foundation of Jilin Province (Project No: 20200708078YY), Jilin Provincial Department of Human Resources and Social Security (Project No: 2023QN16) and gy Foundation of Jilin Province (Project No: 2013QN16).

## CONFLICT OF INTEREST STATEMENT

The authors have no conflicts of interest to declare.

## INFORMED CONSENT STATEMENT

Informed consent was obtained from all subjects involved in the study.

## Supporting information


Appendix S1:

## Data Availability

The data that support the findings of this study are available on request from the corresponding author. The data are not publicly available due to privacy or ethical restrictions.
