# Supplementary material for: Effects of whole nutritional formula foods on nutritional improvement and intestinal flora in malnourished rats
Source: Food Sci Nutr. 2023 Nov 29;12(3):1724–35. doi: 10.1002/fsn3.3865 (PMC10916550; doi:10.1002/fsn3.3865)
Supplement: Supplementary file 1 — Appendix S1: [file FSN3-12-1724-s001.docx]

Supplementary Data 1:

Table A: Formulas for three WNF foods

| Macronutrients | Units | Content（g/100g） | | |
| --- | --- | --- | --- | --- |
|  |  | WNF | FOS | SDF |
| carbohydrate | g/100g | 46.98 | 34.84 | 34.84 |
| whey protein |  | 11 | 11 | 11 |
| [soy](javascript:;) [protein](javascript:;) |  | 10 | 10 | 10 |
| Fat |  | 17.24 | 17.24 | 17.24 |
| α-linolenic acid |  | 2.87 | 2.87 | 2.87 |
| [linoleic](javascript:;) [acid](javascript:;) |  | 0.95 | 0.95 | 0.95 |
| [Fructo-oligose](javascript:;) |  | - | 12.14 | - |
| Ginseng-SDF |  | - | - | 12.14 |
| V_A_ | mg RE/100g | 450 | 450 | 450 |
| V_D_ | μg/100g | 5 | 5 | 5 |
| V_E_ | mg/100g | 10 | 10 | 10 |
| V_K1_ | μg/100g | 36 | 36 | 36 |
| V_B1_ | mg/100g | 1 | 1 | 1 |
| V_B2_ | mg/100g | 0.8 | 0.8 | 0.8 |
| V_B3_ | mg/100g | 8 | 8 | 8 |
| V_B5_ | mg/100g | 3 | 3 | 3 |
| V_B6_ | mg/100g | 1 | 1 | 1 |
| V_B9_ | μg/100g | 180 | 180 | 180 |
| V_B12_ | μg/100g | 2 | 2 | 2 |
| V_C_ | mg/100g | 70 | 70 | 70 |
| [Biotin](javascript:;) | μg/100g | 27 | 27 | 27 |
| Na | mg/100g | 500 | 500 | 500 |
| K | mg/100g | 650 | 650 | 650 |
| Cu | μg/100g | 550 | 550 | 550 |
| Mg | mg/100g | 120 | 120 | 120 |
| Fe | mg/100g | 7 | 7 | 7 |
| Zn | mg/100g | 6 | 6 | 6 |
| Mn | μg/100g | 1500 | 1500 | 1500 |
| Ca | mg/100g | 650 | 650 | 650 |
| P | mg/100g | 300 | 300 | 300 |
| I | μg/100g | 40 | 40 | 40 |
| Cl | mg/100g | 500 | 500 | 500 |
| Se | μg/100g | 25 | 25 | 25 |
